# Towards seasonal forecasting of flood probabilities in Europe using climate and catchment information

**DOI:** 10.1038/s41598-022-16633-1

**Published:** 2022-08-06

**Authors:** Eva Steirou, Lars Gerlitz, Xun Sun, Heiko Apel, Ankit Agarwal, Sonja Totz, Bruno Merz

**Affiliations:** 1 Section Hydrology, GFZ German Research Center for Geosciences, Potsdam, 14473 Germany; 2 Key Laboratory of Geographic Information Science (Ministry of Education), East China Normal University, Shanghai, 200241 China; 3 Columbia Water Center, Earth Institute, Columbia University, New York, NY 10027 USA; 4 Department of Hydrology, Indian Institute of Technology Roorkee, Roorkee, 247667 India; 5 Department of Civil and Environmental Engineering, MIT, Cambridge, 02138 USA; 6 Institute of Environmental Science and Geography, University of Potsdam, Potsdam, 14476 Germany

**Keywords:** Hydrology, Natural hazards

## Abstract

We investigate whether the distribution of maximum seasonal streamflow is significantly affected by catchment or climate state of the season/month ahead. We fit the Generalized Extreme Value (GEV) distribution to extreme seasonal streamflow for around 600 stations across Europe by conditioning the GEV location and scale parameters on 14 indices, which represent the season-ahead climate or catchment state. The comparison of these climate-informed models with the classical GEV distribution, with time-constant parameters, suggests that there is a substantial potential for seasonal forecasting of flood probabilities. The potential varies between seasons and regions. Overall, the season-ahead catchment wetness shows the highest potential, although climate indices based on large-scale atmospheric circulation, sea surface temperature or sea ice concentration also show some skill for certain regions and seasons. Spatially coherent patterns and a substantial fraction of climate-informed models are promising signs towards early alerts to increase flood preparedness already a season ahead.

## Introduction

Can the probability distribution of extreme streamflow in Europe be forecasted based on hydrological information and large-scale climate indices of the preceding season? This is the main question addressed in this work. Flood peaks are influenced by the antecedent catchment wetness and by large-scale atmospheric circulation^1–5^. Such climate-flood linkages lead to periods of above or below average flood peaks and losses^6,7^. In case these variations are substantial and can be predicted a season ahead, they open up additional strategies for flood risk management. They allow early alerts, complementing existing forecasts at shorter time scales, to increase flood preparedness. Examples are the early release of water from reservoirs to increase their flood retention capacity or the procurement of disaster response supplies^8,9^.

The potential of seasonal forecasting of streamflow for water resources management has been explored for irrigation^10^, navigation^11^, and reservoir management^12^, typically within the context of too little water, while its use for too much water is mostly unexplored^9^. Seasonal forecasting is either based on simulation models^13^ or statistical relationships^14^. Here, we apply the latter approach by exploring whether catchment and climate indices can be used as covariates within flood frequency analysis to understand changes in flood peak distributions a season ahead for more than 600 gauges in Europe.

Traditional flood frequency analysis fits an extreme value distribution to the tail of the streamflow observations (block maximum or peak-over-threshold values). This approach is based on the iid (independent, identically distributed) assumption, i.e. all flood events are assumed to be independent realizations of the same distribution. For situations where the flood peak distribution varies significantly with climate or catchment state, the assumption of a single distribution for all flood events can be relaxed. In this case, the flood peak distribution can be conditioned on covariates describing the climate or catchment state. For some regions, this approach (called conditional or climate-informed flood frequency analysis) has been found to be superior to the classical, unconditional flood frequency analysis^15–19^. If the covariate describes the catchment or climate state of the season ahead, the conditional approach can be used for seasonal forecasts of flood peak distributions.

For Europe, several studies have explored the benefit of the conditional flood frequency approach. Villarini et al.^20^ quantified the variation of flood peaks for Austria as function of North Atlantic Oscillation (NAO). López and Francés^21^ and Machado et al.^22^ used climate indices, and a reservoir index, for describing the time-variation of flood peaks in the Iberian Peninsula. A few studies used precipitation indices as covariates for flood frequency analysis in the United Kingdom (UK)^23–26^. Bertola et al.^27^ explored if flood changes in Upper Austria can be attributed to potential atmospheric, catchment and river system drivers, using precipitation, land use and reservoir indices. Finally, Steirou et al.^19^ identified links between seasonal flood probabilities and large-scale atmospheric indices for entire Europe. They found that the conditional approach was superior compared to the traditional flood frequency analysis for a large fraction of stations. These stations were not randomly distributed across Europe but formed spatial patterns that could be explained by the effects of different atmospheric circulation patterns (represented by specific climate indices) on flood generation processes. Hence, these results suggest significant climate-flood linkages for Europe.

However, these studies have only investigated contemporaneous relationships between climate indices and extreme streamflow, i.e. flood peaks were related to the climate of the same period. This approach allows to explain to which extent the time-variation of flood peak distributions can be explained by climate variability, but it does not allow to forecast the change in the flood peak distribution for the coming season. To explore the potential of seasonal forecasting of flood probabilities for Europe, we extend the approach of a previous study^19^ by investigating the possibility of lagged relations between catchment or climate state and flood peaks.

To select promising covariate variables, we build on the experience of forecasting streamflow in Europe. The skill of streamflow forecasts arises from two sources: the catchment state prior to the prediction period, and the potential to forecast future climatic conditions. Previous studies for Europe show that variables representing the wetness of the catchment can serve as skilful predictors of seasonal mean and maximum discharge^9,28^. Furthermore, the European climate is predictable to some extent, since the lower boundary conditions, i.e. the state of the land and sea surface, influence the atmospheric circulation on a time scale of several months. For instance, Sea Surface Temperature (SST) anomaly patterns in the North-Atlantic domain, snow cover variations over Eurasia and sea ice fluctuations in the Arctic have been shown to significantly influence the European precipitation climate^29–31^.

In this study, we adopt covariates for the season ahead from both categories. Catchment state proxies will hereinafter be referred to as “catchment covariates”. They include local hydrometeorological variables, such as precipitation, and large-scale climate indicators, such as NAO. The latter variables would be considered climate covariates, if contemporaneous relationships were examined. However, as they are derived for the period preceding the flood season, they rather serve as proxy for the catchment wetness prior to the flood season than as direct proxy for future climate. Variables that act on longer time scales, such as sea ice variations, have a higher potential to forecast future climatic conditions and consequently streamflow, and will be referred to as “climate covariates”. Based on the consideration of both, catchment and climate predictors, our study investigates all relevant sources of hydrological predictability in the framework of a conditional flood frequency analysis. The exploratory approach allows to evaluate the predictive skill of various variables for the entire European continent for the first time and has the potential to serve as a basis for the development of comprehensive models for the forecast of seasonal flood probabilities.

## Data and indicators representing catchment and climate state

Daily discharge time series are provided by the Global Runoff Data Centre^32^. We consider in total 649 records covering mostly north, north-western and central Europe. The study covers the period 1950 to 2016, and only records of lengths of at least 50 years are considered. For more details about station selection, the reader is referred to a previous study^19^. A wide range of catchment and climate covariates could be used for seasonal forecasting of flood probabilities. We constrain our study to covariates that are easy to derive and include in an operational forecasting scheme when catchment boundaries and a detailed Digital Elevation Model (DEM) are not available. All covariates were obtained from open climate datasets or are easy to extract from the streamflow records.

We classify our predictor data sets into (a) catchment covariates, that indicate the state of the catchment wetness at the beginning of the forecasting season, and (b) climate covariates, that provide a certain forecast potential for the climate conditions of the upcoming season.

### Catchment covariates

To describe the catchment state prior to the flood season, we include precipitation (abbreviation “Prec” is used in figures) and temperature (abbreviation “Temp” is used in figures) as potential predictors. Season ahead precipitation directly increases catchment wetness, while season ahead temperature affects catchment state via evapotranspiration and snowmelt. Monthly temperature and precipitation data is derived from the gridded CRU TS4.02 dataset^33^ and extracted for the location of the gauges. While the catchment average of temperature and precipitation might be a more representative predictor than the value of the specific CRU grid cell, we assume that our pragmatic approach well represents the inter-annual climate variability. We include streamflow prior to the flood season (abbreviation “Disc” is used in figures) which is a widely used indicator representing the effect of catchment state on flooding^4,34^.

Besides these local catchment covariates, we consider large-scale atmospheric circulation modes, provided by the NOAA Climate Prediction Center as proxies for the preceding climate and thus the catchment state. The hydrological condition of European catchments is closely linked to prevailing circulation characteristics, which drive the regional moisture fluxes and the spatio-temporal precipitation variability at a continental scale. Particularly the North Atlantic Oscillation (NAO), but also the East-Atlantic/Western-Russia (EAWR), the Scandinavian (SCA), the East-Atlantic (EA) and the Polar-Eurasian (POL) patterns have been shown to modulate flood probabilities in Europe ^5,19,35^. Due to the hydrological memory effect, an influence on flood frequencies may be also expected for subsequent periods. For instance, water stored in the snowpack, in soils or groundwater aquifers prior to the flood season will be released from the catchment or will influence catchment response to precipitation during the forecast period.

All variables representing catchment state are aggregated to three-month averages to represent the average conditions of the season ahead. Tests with monthly catchment predictors were conducted in a feasibility study; however, no significant influence of the aggregation period on the forecast results has been detected. All covariates are tested for their forecast potential with lead times between 0 and 2 months, for instance, flood frequency time series for winter (Dec–Jan–Feb) are related to predictor records for Sep-Oct-Nov (lead time 0), Aug-Sep-Oct (lead time 1) and Jul-Aug-Sep (lead time 2).

### Climate covariates

To evaluate the potential of seasonal climate predictions for assessing flood probabilities, we test the skill of covariates traditionally employed in seasonal climate forecasting routines. In particular, we make use of the first five principal components (PCs) of de-trended SSTs over the North-Atlantic domain (10–70° N, 80° W–20° E) for the period 1950–2017, derived from the ERSST v03 dataset^36^ (hereinafter SST1-5, see Figs. S1, S2 for details). These PCs represent the temporal variability of the spatial distribution of sea surface temperatures over the Northern Atlantic Ocean, which have been shown to drive the development of atmospheric circulation patterns over Europe. Due to the low-frequency variability of the sea surface temperatures, the patterns provide a certain potential for the forecast of pressure patterns and thus of the European precipitation climate during the season ahead. While the first PC (SST1) is characterized by a uniform temperature distribution which is directly related to the annual temperature cycle, the following PCs resemble well known patterns such as the Atlantic Multidicadal Oscillation (SST2) and the North Atlantic Tripole (SST4, see Fig. S1). Particularly the latter has been shown to influence the state of the NAO during winter season^30,37^, which is also indicated by significant correlations between the PC loadings and the NAO index (Fig. S2). Furthermore, we use Sea Ice Concentration (SIC) between 70 and 80° N, 30 and 105° E, which has been identified as an impontant driver of the midlatitude circulation^38^ and as a suitable predictor for winter precipitation anomalies for Europe^31^. As these climatic predictors represent the boundary conditions of the climate system at a specific point in time, which might affect the development of the atmospheric circulation in the near future, no temporal aggregation is conducted. As for catchment state variables, the skill of climatic covariates is investigated for lead times between 0 and 2 months, i.e. the forecast skill for winter (Dec–Jan–Feb) flood probabilities is analyzed for covariates in Nov (lead time 0), Oct (lead time 1) and Sep (lead time 2).

In total, we investigate the skill of 14 covariates to forecast flood frequencies: eight catchment state predictors (three local hydrometeorological variables and five variables representing large-scale circulation) and six climate predictors (five variables representing SSTs and one SIC variable). The number of stations examined varies between 570 and 600 for the different combinations of seasons and lead times, because data gaps, either in the streamflow or the covariates, lead to times series shorter than 50 years in several cases. For each station, the common period of the maximum streamflow time series and examined covariates is considered.

## Methods

The Generalized Extreme Value distribution (GEV) is used for the modeling of seasonal streamflow maximum values. The model is local and is fitted independently to each streamflow time series. A Bayesian framework is used for inference. It allows the inclusion of prior knowledge in the modeling procedure and uncertainty handling in a rigorous way^39^. The posterior probability density function (pdf) of the parameter vector is computed based on the Bayes theorem as follows:

1 $$f\left( {{\varvec{\theta}}{|}{\varvec{Y}}} \right) \propto f({\varvec{Y}}|{\varvec{\theta}})f\left( {\varvec{\theta}} \right),$$

where *f(***θ***)* is the prior pdf of the distribution parameters, ***Y*** is the vector of streamflow observations at a specific site and *f(***Y***|***θ***)* the likelihood function:

2 $$f\left({\varvec{Y}}|{\varvec{\theta}}\right)=\prod_{t}f\left(Y(t)|{\varvec{\theta}}\right)$$

For the conditional distribution, parameters are variable in time, and thus, the vector parameter ***θ*** in the above equations is replaced with ***θ(t)***.

Conditional and unconditional models are fitted independently of each other, and conditional models are limited to a single covariate. For instance, the location parameter of the flood GEV distribution is assumed to be a linear function of the average NAO value in the season ahead. Models with more than one covariate at a time are also possible, but since the number of different models is already very high, given 14 covariates, four seasons, and three lead times, and since several covariates are significantly correlated (some examples are shown in Fig. S2), we limit our study to the most parsimonious case.

The classical GEV is given as follows:

3 $$Y\left(t\right)\sim GEV(\mu , \sigma , \xi )$$

It comprises three parameters: a location parameter μ, a scale parameter σ and a shape parameter ξ. A preliminary analysis showed that both the location and scale parameters vary with covariate values. Thus, for the conditional/climate-informed models, Eq. (3) takes the form:

4 $$Y\left(t\right)\sim GEV(\mu (t), \sigma (t), \xi )$$

Both parameters are modeled as a linear function (without error term) of the examined covariates as follows:

5 $$\mu \left(t\right)={\mu }_{0}+{\mu }_{1}x(t)$$

6 $$\sigma \left(t\right)={\sigma }_{0}+{\sigma }_{1}x(t)$$

where *μ(t)* is the varying location parameter, *μ*_*0*_ the location intercept, *μ*_*1*_ the location slope and *x(t)* the single covariate examined. Similarly, *σ(t)* is the varying scale parameter, σ_0_ the scale intercept and σ_1_ the scale slope. The shape parameter is held constant as its estimation includes large uncertainties^40^. Consequently, our conditional/climate-informed GEV comprises five parameters (*μ0, μ1, σ0, σ1, ξ*) versus the three parameters (*μ, σ, ξ*) of the unconditional GEV. These modeling choices are in line with previous approaches^16,18,24^. Non-informative uniform priors are used for the intercept and slope of the location and scale of the conditional model and for the location and scale of the unconditional model. The shape parameter in both cases is assigned a normal prior distribution with a mean of 0.093 and standard deviation of 0.12, based on results of previous studies^19,40^.

Climate-informed models are compared to the unconditional GEV in a pairwise way, and the preferred model among the two is selected. The conditional model needs to meet simultaneously two criteria in order to be selected over the unconditional case (a more detailed description can be found in Steirou et al.^19^). Firstly, the Deviance Information Criterion (DIC)^41^ is employed which assesses each model’s goodness-of-fit and adds a penalty term for model complexity, thus favouring simpler models. Climate-informed models with lower DIC value than the unconditional model are preferred in this step. Secondly, the climate-informed model is chosen over the unconditional distribution only when the 90% credibility interval of the location or scale slope does not contain the zero value. No direct model comparison between conditional models is performed during model selection as we are interested in all climate-informed cases that are a potential improvement versus the classical GEV.

## Results and discussion

To evaluate whether the selected catchment and climate covariates have the potential to be used in seasonal forecasting of flood probabilities, the percentage of stations with climate-informed models preferred over the unconditional model is assessed. The results are given for the location and scale parameter, so that the effect of the catchment and climate covariates on the mean and variability of the flood distributions can be assessed separately. Results are also given for selected European regions to identify covariates that may have forecasting skill but at a smaller spatial scale. Five regions with substantial differences in their flood seasonality are examined: Scandinavia; North Germany—Netherlands; United Kingdom—Ireland; the Alpine region; Eastern Europe (Fig. S3).

**Figure 1:**
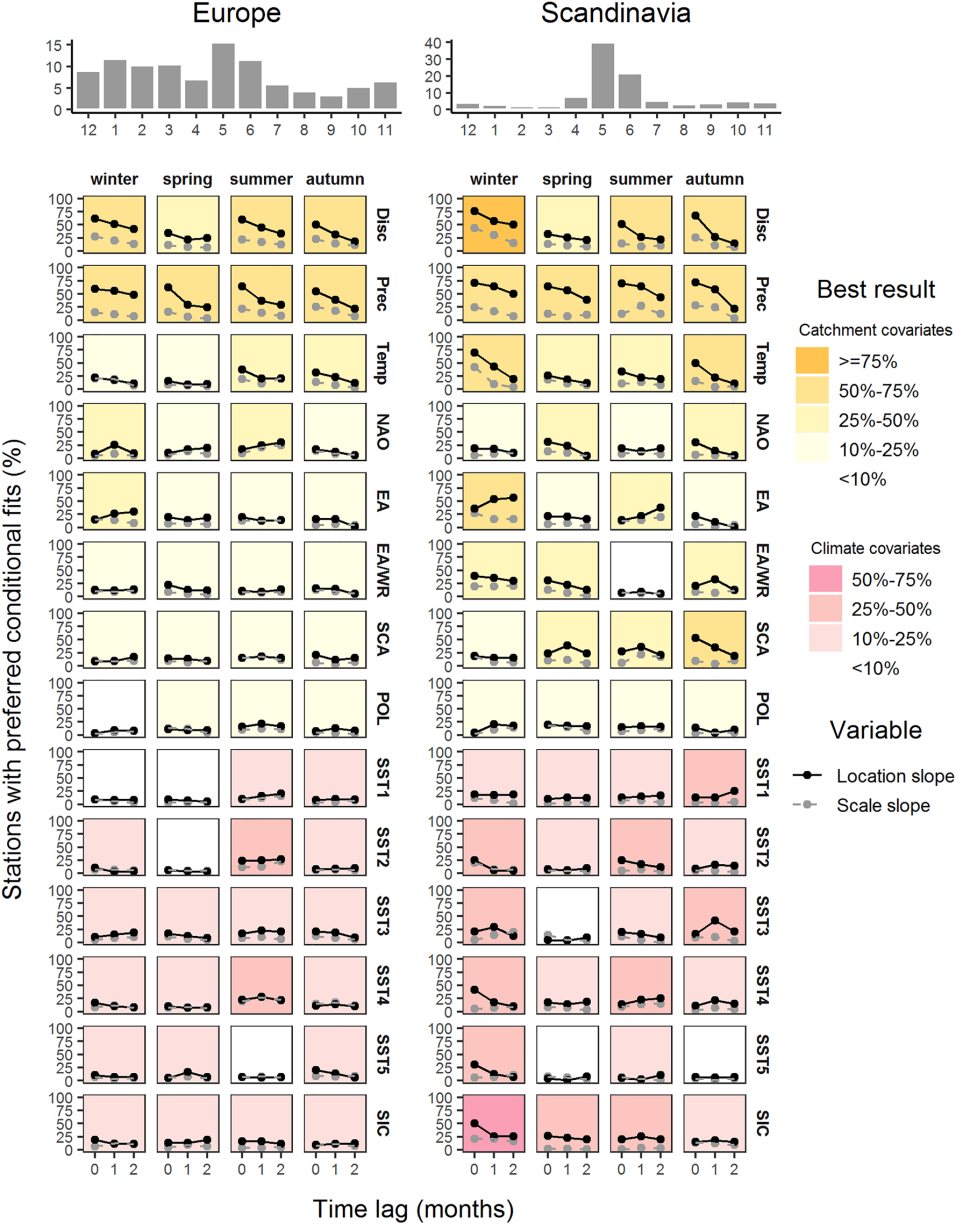
Summary of preferred conditional models for all 14 covariates, all seasons and the three lead times. Results are shown for the whole of Europe (left) and Scandinavia (right). In the main panel, the solid (dashed) lines show the percentage of stations for which the respective covariate has an effect on the location (scale) slope. The legend colour refers to the best result (highest station percentage) with an effect on the location slope among the three lead times. Catchment and climate covariates are differentiated by color palette, orange and pink, respectively. On top of the main panel, flood seasonality for the two regions is given as histogram of the percent monthly relative frequency of annual maximum streamflow. The figure was created using the R package ggplot2, version 3.2.0.

**Figure 2:**
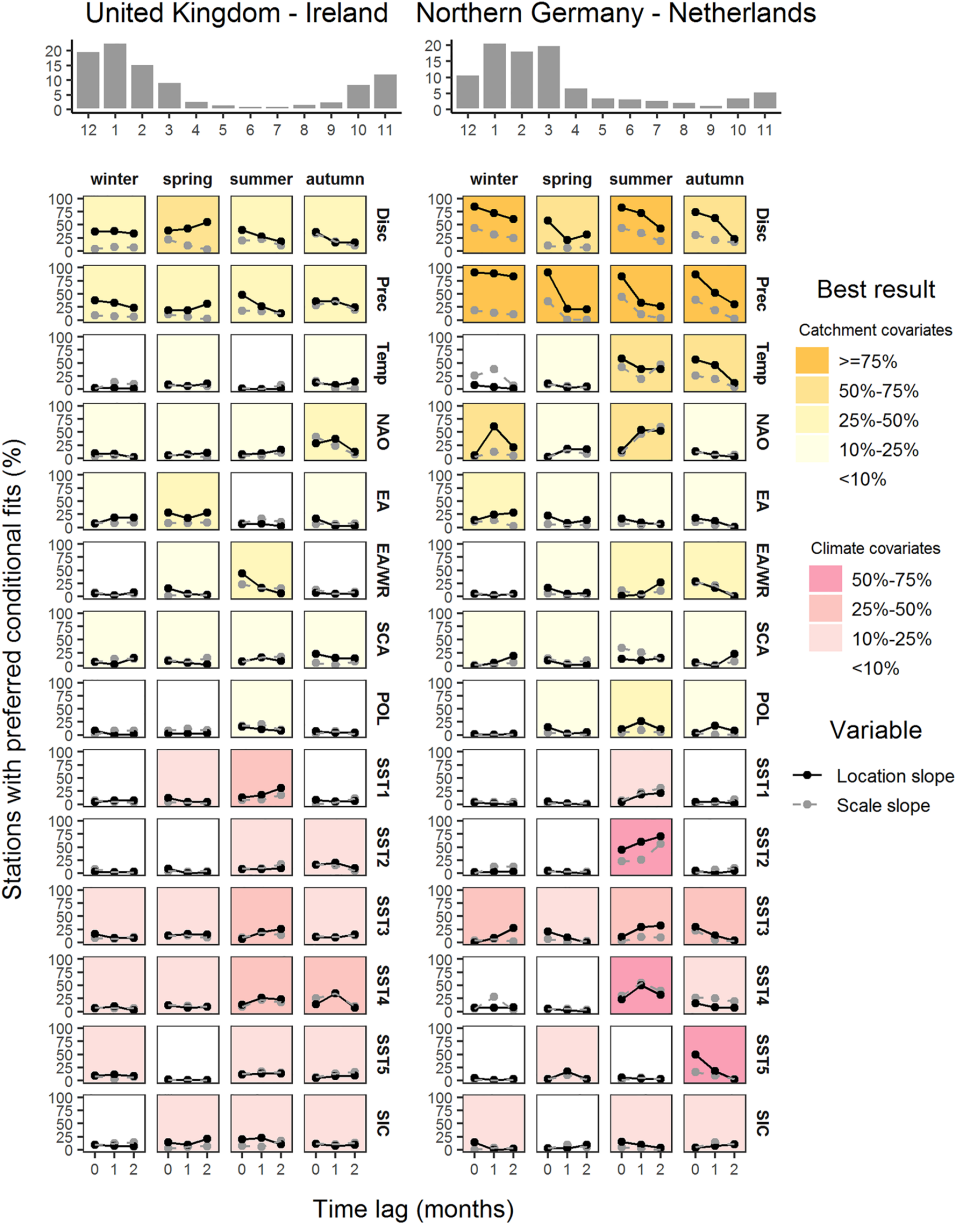
Same as Fig. 1 but for the sub-regions United Kingdom–Ireland (left) and Northern-Germany–Netherlands (right).

The fraction of climate-informed models preferred over the unconditional models varies across covariates, seasons, lead times, GEV parameters and regions (Figs. 1, 2, S4). For instance, for Europe and the location parameter, 62% of stations show preferred climate-informed models for the winter season (Dec–Jan–Feb), when the average streamflow of the season ahead (Sep–Oct–Nov, lead time 0) is used as covariate for the GEV location parameter (Fig. 1). This high fraction drops to 9% when SIC serves as covariate for autumn maxima.

Despite large variations across different combinations of covariates, seasons, lead times and regions, cases with significant location slopes are much more common than significant scale slopes. Hence, changes in the mass of the flood distributions are more substantially linked to season-ahead covariates than changes in the variability of flood distributions. Previous studies in Europe and elsewhere have found both significant and negligible effects of climate covariates on the scale of the flood distribution^16,19,20^. Below we discuss percentages of stations for models with significant effects on the location parameter.

For most combinations of seasons and lead times, catchment covariates show a higher potential for seasonal flood forecasting than climate covariates. This result holds for the whole of Europe and all the explored regions and shows the importance of initial catchment wetness for flood forecasting, as also suggested by hydrological modeling^13^. Among the catchment covariates, antecedent precipitation and antecedent discharge are the best covariates, again for entire Europe and the individual regions. Similar results for these two covariates suggest that they are closely related proxies for season-ahead catchment state. However, they show distinct differences in selected cases. For instance, winter precipitation has a high potential to forecast maximum streamflow in spring in the Alps and in Eastern Europe, while winter discharge has a low potential (Fig. S4). This difference can be explained by the role of snow in these regions. Winter precipitation often falls as snow and increases the water availability during the following spring. During the winter season, the contemporaneous effect of precipitation on streamflow is marginal as the water is stored as snow.

**Figure 3:**
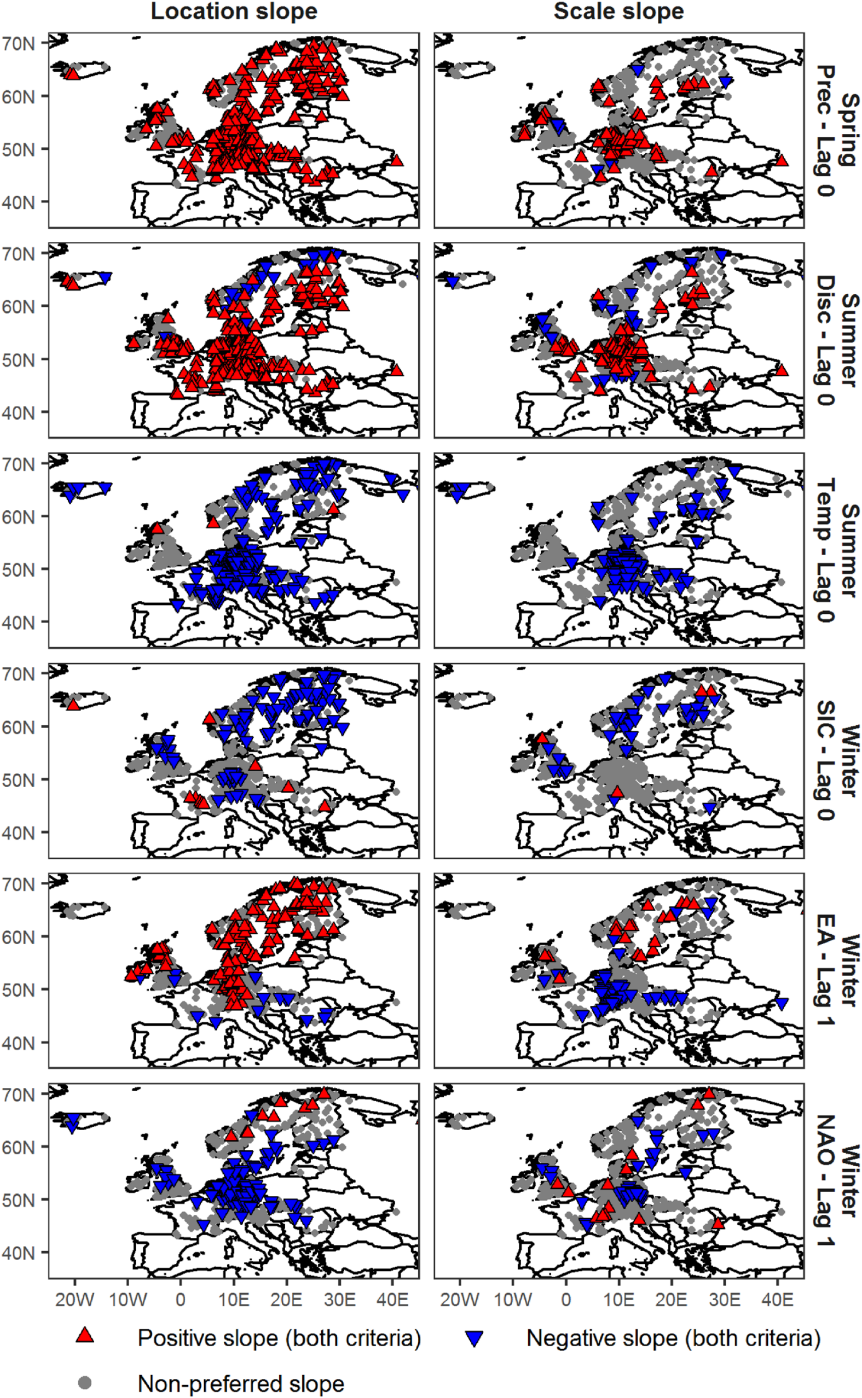
Spatial patterns of preferred climate-informed models for GEV location and scale for selected combinations of seasons, covariates and lead times. Preferrence is determined by two criteria: lower DIC value and the 90% credibility interval of the slope for the specific GEV parameter does not contain the zero value. Grey circles show gauges where no effect is found on the location (left) or scale (right) slope. Triangles show gauges where the examined covariate has an influence on the respective GEV parameter. Upward/red (downward/blue) triangles show positive (negative) association between season-ahead covariate and GEV parameters. The figure was created using the R package ggplot2, version 3.2.0. Country borders source: https://thematicmapping.org/.

The third local catchment covariate, season-ahead temperature, shows low potential for the forecast of flood probabilities with a few exceptions. For instance, autumn temperature is a very good predictor (70% of stations show preferred climate-informed models) for winter maximum streamflow in Scandinavia, and summer temperature has a high potential for forecasting maximum streamflow in autumn in Scandinavia and North Germany–Netherlands (Figs. 1, 2). The relation between temperature and maximum streamflow is negative (Fig. 3), i.e. a warmer season ahead tends, possibly via higher evapotranspiration, to decrease the flood probabilities. However, from the perspective of flood risk management these cases are not very relevant as these regions show little flood activity in these seasons (see flood seasonality histograms in Figs. 1 right and 2 right).

The large-scale catchment indices, such as the average NAO value for the season ahead, show a low potential for seasonal flood forecasting for the whole of Europe (Fig. 1 left). Again, there are a few exceptions for some regions. For Scandinivia, autumn EA and summer SCA are good predictors for forecasting winter and autumn maximum streamflow, respectively (Fig. 1 right). NAO has some potential for winter and summer maximum streamflow in North Germany–Netherlands (Fig. 2 right), and POL has some potential for summer maximum streamflow in Eastern Europe (Fig. S4 right). However, in most cases, relations do not persist across different regions and different lead times, and thus should be considered with caution.

The six climate covariates show a low potential for seasonal flood forecasting for the whole of Europe (Fig. 1 left), but some potential at the regional scale. For example, November SIC significantly affects winter maximum streamflow for 50% of all stations in Scandinavia, which can be explained by the modulation of the winter state of NAO by the northern hemispheric snow coverage in preceding autumn^30,37^. A similar potential is given for some combinations of seasons and climate indicators for the Alps, and North Germany–Netherlands. However, these cases are not of high importance for flood risk management due to their low flood activity. Of higher relevance is the potential of February SST3 for spring floods (62% preferred climate-informed models) and May SST4 for summer floods (58% preferred climate-informed models) in Eastern Europe, which indicates an influence of the SST patterns on the regional circulation and thus the local precipitation climate during spring and summer season. While the teleconnection between the North Atlantic SSTs (in particular the North Atlantic Tripole) and the state of NAO during winter and spring seasons have been widely discussed in literature^30,37^, the relationships between the North Atlantic SST distribution and the flood activity during summer season is rather complex since high precipitation events during summer are usually triggered by local-scale convection. Although some progress has been made to forecast summer weather patterns over Europe based on the preceding oceanic forcing^42^, the exact mechanisms beyond the observed relationships require further research.

Among all covariates examined, we observe a higher forecasting potential for the local catchment variables in comparison to large scale covariates, catchment or climate. This can be can be explained by their more direct link to maximum streamflow. Precipitation and streamflow anomalies in the season ahead can directly affect flood probabilities, whereas anomalies in large-scale indicators, such as NAO, can affect flood probabilities only indirectly by modulating catchment precipitation, temperature and thus streamflow.

Overall, the forecasting potential decreases with lead time, in agreement with recent streamflow forecasting studies in Europe using simulation models^9,43^. The season/month immediately preceding the target streamflow season shows, in most cases, a higher fraction of preferred climate-informed models compared to higher lead times. The effect of lead time is a bit more variable for climate covariates. In some cases, the potential persists or even increases with higher lead times, which displays the complexity of climatic teleconnections and shows that the selection of feasible climate predictors requires a regional and seasonal specific exploration, as also suggested by Ionita et al.^14^.

Comparing all regions, we find the lowest potential for seasonal flood probability forecasting for UK–Ireland. Autumn and winter are the seasons with the highest flood activity in this region. For these two seasons, antecedent streamflow is the best covariate, with summer and autumn discharge affecting autumn and winter flood probabilities for 36% and 37% of stations, respectively (Fig. 2 left).

Figure 3 illustrates the spatial patterns of preferred climate-informed models for the whole study area and selected cases of covariates and lead times. In all cases, the stations with preferred climate-informed models form regional patterns. For instance, higher autumn EA is linked to higher winter maximum streamflow for large parts of Central and Northern Europe, but it is linked to lower winter maximum streamflow for Southern and Eastern Europe. These spatial patterns are different for the location and scale parameters. For Germany, for example, higher autumn EA tends to increase the location parameter of the flood distribution in winter, but it tends to decrease its scale. The spatial coherence of the climate-informed models that are fitted on a gauge-by-gauge basis demonstrates that the identified relations between season-ahead covariates and flood probabilities are a consequence of regionally varying flood generation processes.

For selected stations, covariates and seasons, observed seasonal streamflow is further compared with obtained flood estimates for probability of exceedance 0.1 (corresponding to the 10-year return period of the classical/unconditional case). An example of this local analysis is shown in Fig. S5 for a station at the Oder river. Several but not all winter discharge peaks are captured by the seasonal forecast based on autumn precipitation. In summary, the obtained results vary depending on the station location and the combination of season and covariates, suggesting that a more regional and seasonal specific exploration may be required to further improve the forecasting skill.

Deprite the promising results, which indicate a high forecasting potential for some European regions, our study has a number of limitations. Firstly, the station density is very different across Europe. For some regions with low density, e.g. Eastern Europe, our results are less trustworthy. Further, uninformative priors are used for the location and scale intercept and slope. While more informative priors would presumably improve the fit of the models and their forecast potential, the scale of the study (over 200,000 models due to the large number of stations and combinations of covariates, seasons and lead times) would have made the inclusion of true prior information a herculian task. Because of the large number of models investigated, our exploratory study derives only results for a single covariate for each season. In order to further improve the model skill, the simultaneous consideration of suitable climate and catchment predictors in a single (bivariate) model to investigate is certainly promising (see also similar argumentation in Robertson and Wang^44^). Finally, covariates were chosen so that they are easily extractable without the need for catchment boundaries. An improvement could result from using catchment averages for antecendent precipitation and temperature instead of grid data which might be much less representative for the catchment state.

## Conclusions

While the benefit of seasonal forecasting using statistical approaches has been investigated for a number of purposes, seasonal forecasting of flood probabilities has received little attention so far. Here, we explore whether the inclusion of catchment or climate information of the season ahead improves the estimation of the flood probability distribution for around 600 streamflow stations across Europe. We find that such climate-informed models are preferred over the classical, unconditional approach for a substantial number of stations. These stations with significant climate-flood linkages form spatially coherent patterns, a sign that these linkages emerge from the interaction of the large-scale climate and land surface processes during flood generation.

The high number of preferred climate-informed models suggests that there is a substantial potential for seasonal forecasting of flood probabilities in Europe. This potential varies across seasons, regions and covariates. Overall, the season-ahead catchment wetness, in particular antecedent precipitation and discharge, shows the highest potential to forecast the flood probability. Climate indices, based on atmospheric circulation, sea surface temperature or sea ice concentration, are less skillful. This is explained by the fact that they do not affect flood probabilities directly; instead they affect flood probabilities by altering the large-scale climatic circulation and consequently the regional precipitation regimes and related catchment processes, such as evapotranspiration, water storage and runoff generation. Future work should explore whether these promising results yield enough benefit for using climate-informed models in an operational forecasting context. To this end, the bias and uncertainty of the forecasts, and the variations of the flood peak distributions as function of covariates, need to be compared against the specific enduser demands.

## Supplementary Information

Supplementary Figures.

## Data Availability

The GRDC discharge dataset was obtained from the Global Runoff Data Centre, 56,068 Koblenz, Germany (https: //www.bafg.de/GRDC/EN, last access: October 2017), and is available upon request. Time series of monthly circulation indices were retrieved from the Climate Prediction Center (CPC) of the National Oceanic and Atmospheric Administration (NOAA) and can be accessed through http://www.cpc.ncep.noaa.gov/data/teledoc/telecontents.shtml (last access: May 2017). Gridded temperature and precipitation data was extracted from the CRU TS4.02 dataset from the climatic research unit (CRU, https://crudata.uea.ac.uk/cru/data/hrg/, last access: April 2019) of the University of East Anglia. Sea Surface Temperature data was extracted from the ERSST v03 dataset (psl.noaa.gov/data/gridded/data.noaa.ersst.v3.html, last access: April 2019). The Sea ice concentration time series was derived from the Nimbus-7 SMMR and DMSP SSM/I–SSMIS passive microwave dataset provided by the National Snow and Ice Data Center (http://nsidc.org/data/nsidc-0051) and was subsequently processed as analyzed in Kretschmer et al.^38^. This processed time series is available upon request. Country borders used in the analysis were downloaded from https://thematicmapping.org/.
