# Supplementary material for: Towards seasonal forecasting of flood probabilities in Europe using climate and catchment information
Source: Sci Rep. 2022 Aug 6;12:13514. doi: 10.1038/s41598-022-16633-1 (PMC9357046; doi:10.1038/s41598-022-16633-1)
Supplement: Supplementary file 1 — Supplementary Figures. [file 41598_2022_16633_MOESM1_ESM.pdf]

Supplementary Information

Towards seasonal forecasting of flood probabilities in Europe using climate and catchment information

Eva Steirou[1], Lars Gerlitz[1], Xun Sun[2,3], Heiko Apel[1], Ankit Agarwal[1,4], Sonja Totz[5] and Bruno Merz[*,1,6]

[1]Section Hydrology, GFZ German Research Center for Geosciences, Potsdam, 14473, Germany.

[2]Key Laboratory of Geographic Information Science (Ministry of Education), East China Normal University, 200241, Shanghai, China.

[3]Columbia Water Center, Earth Institute, Columbia University, New York, NY 10027, USA.

[4]Department of Hydrology, Indian Institute of Technology Roorkee, 247667, India

[5]Department of Civil & Environmental Engineering, MIT, Cambridge, 02138, USA

[6]Institute of Environmental Science and Geography, University of Potsdam, Potsdam, 14476, Germany

[*]Corresponding author: Bruno Merz (bmerz@gfz-potsdam.de)

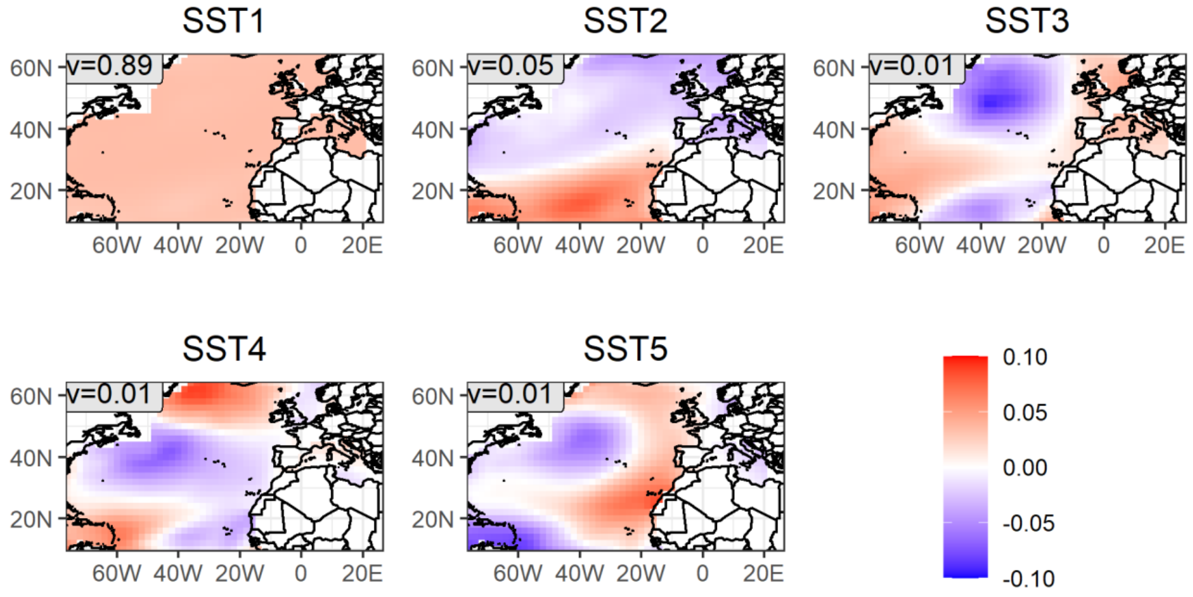

Figure S1. Spatial fields of the first five principal components of monthly mean sea surface temperatures for the North Atlantic (10-70°N, 80°W-20°E) derived from the ERSST v03 dataset [36]. The gray box displays the explained variance of each principal component. The figure was created using the R package ggplot2, version 3.3.6. Country borders source: https://thematicmapping.org/.

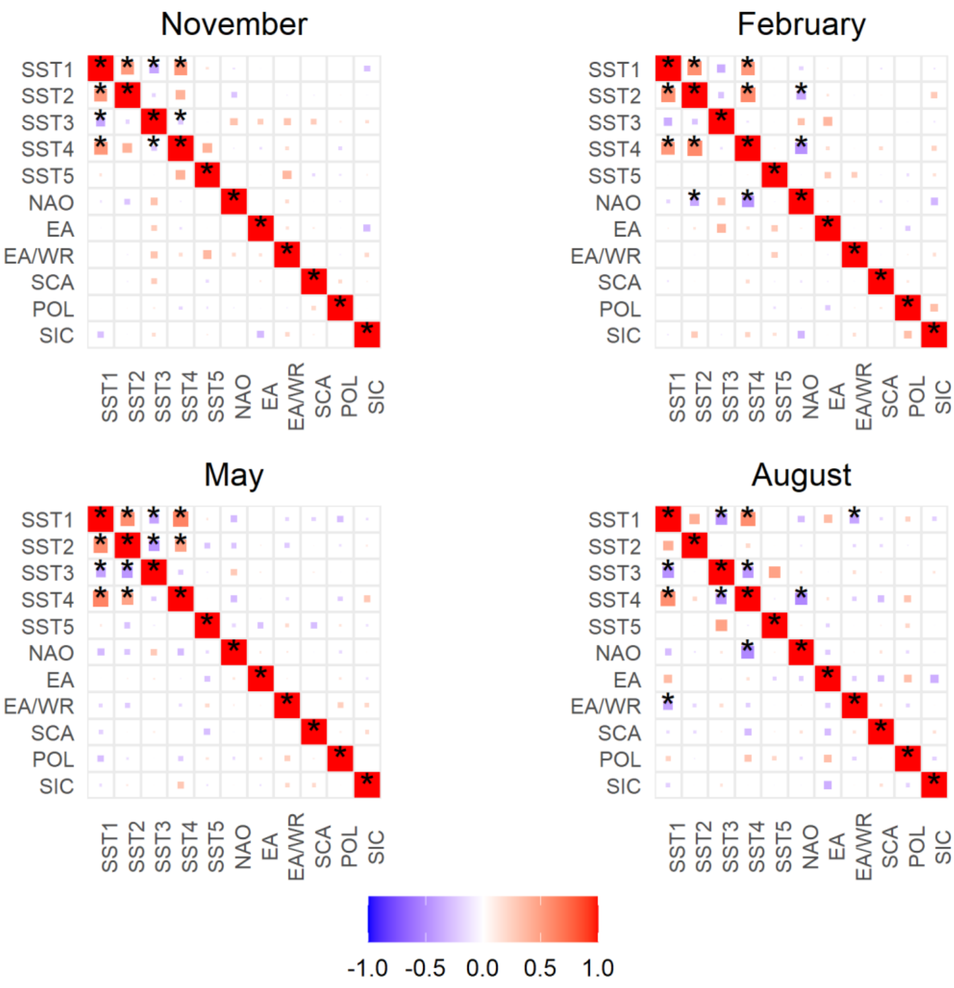

Figure S2. Correlations between all non gauge-specific covariates considered in this study for selected months. The common period of all covariates was used for this calculation. Significant results at the 0.01 level are marked with asterisk. The figure was created using the R packages ggplot2, version 3.3.6, and ggcorrplot2, version 0.1.2 (https://github.com/caijun/ggcorrplot2).

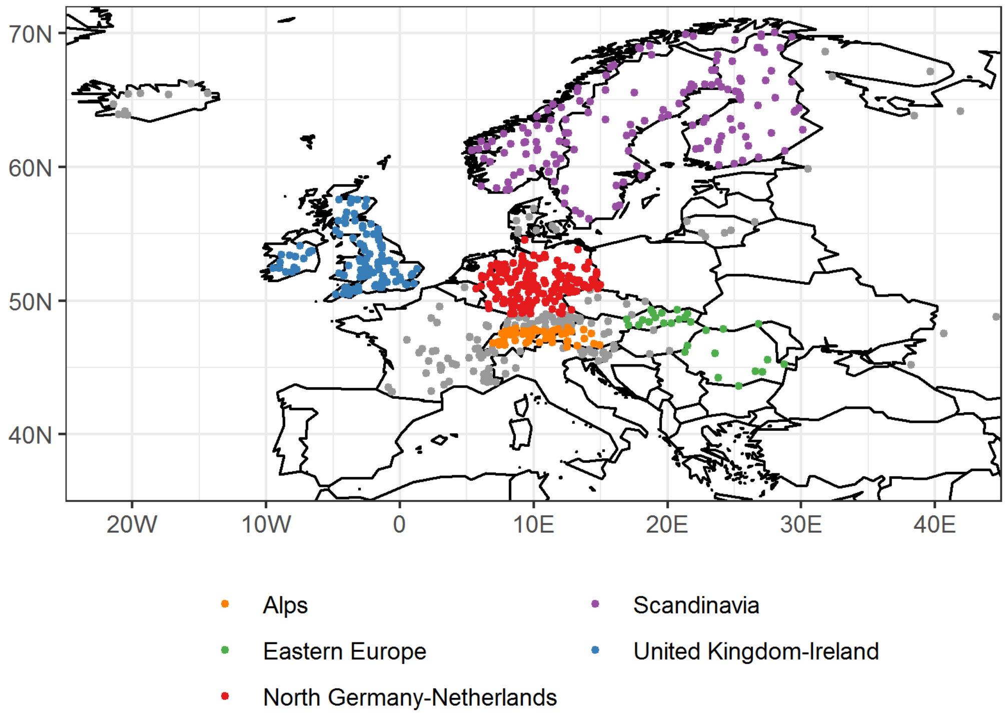

Figure S3. Locations of streamflow stations that were considered for the five regions examined separately. The selection was based on areas with distinct flood seasonality. Grey color indicates stations that were examined only at the European level. The figure was created using the R package ggplot2, version 3.2.0. Country borders source: https://thematicmapping.org/.

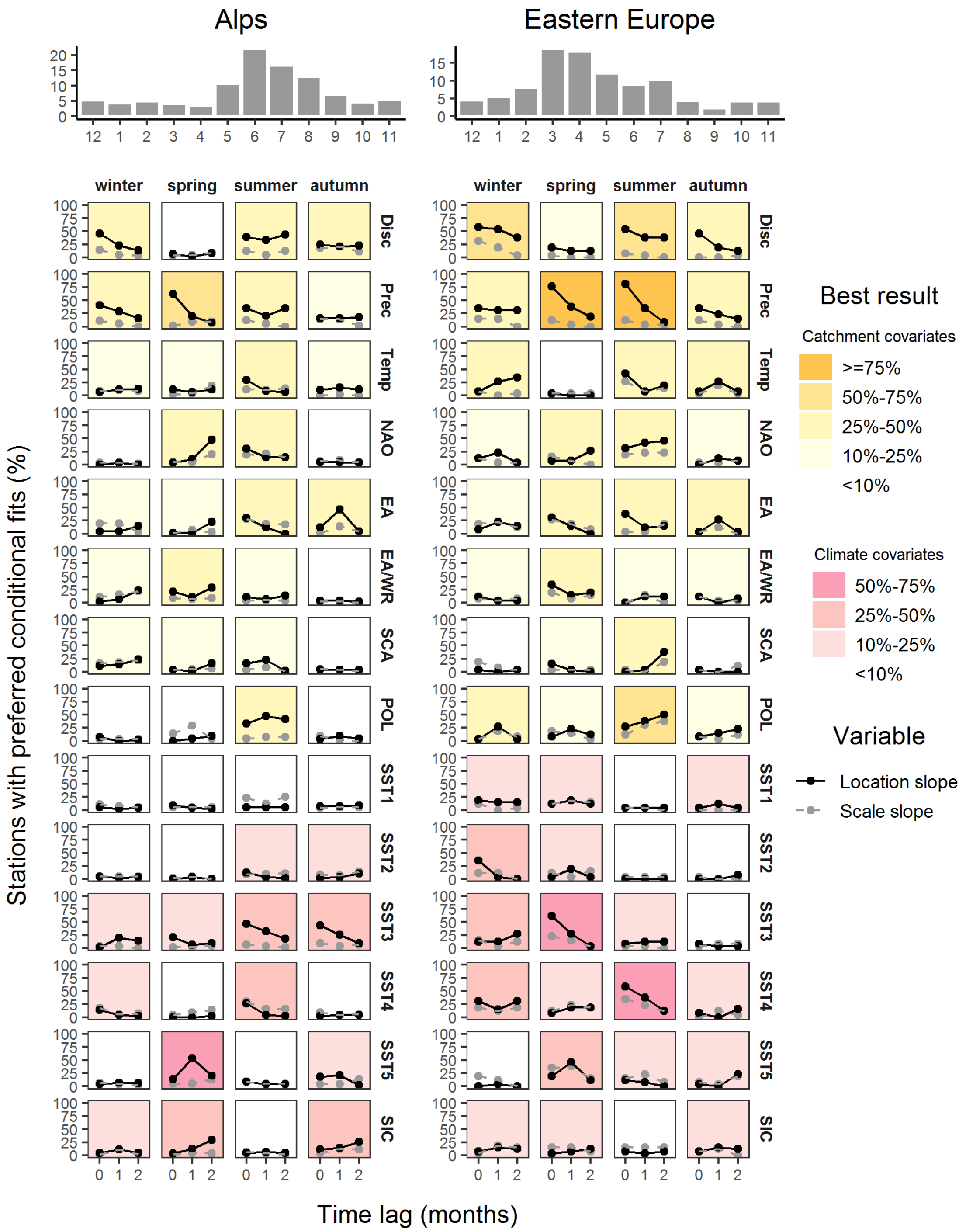

Figure S4. Same as Fig. 1 but for the sub-regions Alpine region (left) and Eastern Europe (right). The figure was created using the R package ggplot2, version 3.2.0.

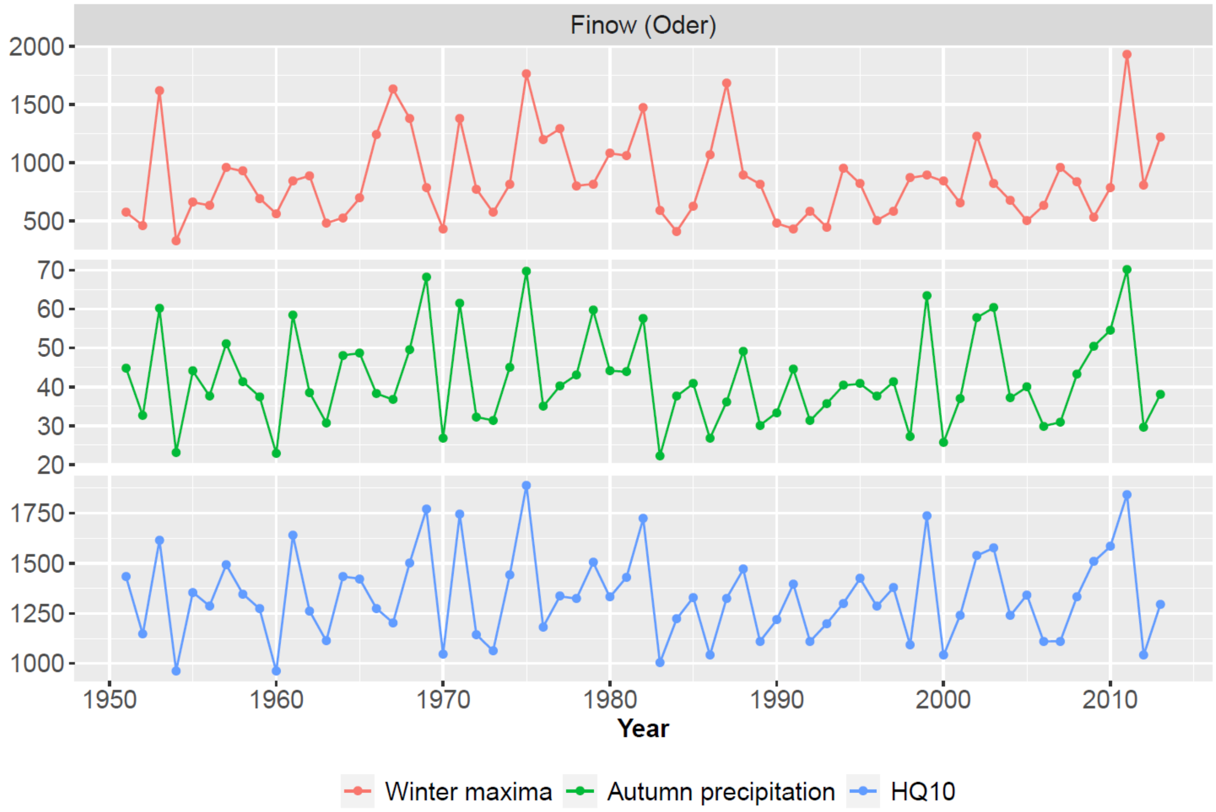

Figure S5. Time series at the station Finow (Oder river). Upper panel: Observed maximum winter streamflow [m^3s^{-1}]; middle panel: autumn precipitation [mm] used as covariate in the climate-informed flood frequency model; lower panel: flood peaks forecasted for winter for a probability of exceedance 0,1 (corresponding to a 10-year return period for the classical/unconditional GEV) [m^3s^{-1}]. The figure was created using the R package ggplot2, version 3.3.6.
